# Merkel cell polyomavirus T-antigens regulate *DICER1* mRNA stability and translation through HSC70

**DOI:** 10.1016/j.isci.2021.103264

**Published:** 2021-10-14

**Authors:** Jiwei Gao, Hao Shi, C Christofer Juhlin, Catharina Larsson, Weng-Onn Lui

**Affiliations:** 1 Department of Oncology-Pathology, Karolinska Institutet; BioClinicum, Karolinska University Hospital, 171 64 Solna, Sweden; 2 Department of Pathology and Cancer Diagnostics, Karolinska University Hospital, 171 64 Solna, Sweden

**Keywords:** Immunology, Immune response, Virology

## Abstract

Merkel cell carcinoma is an aggressive skin malignancy, mostly caused by Merkel cell polyomavirus (MCPyV). MCPyV T-antigens can induce mature microRNA expressions through the DnaJ domain, but its underlying mechanism is still unknown. Here, we report that the T-antigens induce protein expression and mRNA stability of DICER1, a key factor in microRNA biogenesis, through heat shock cognate 70 (HSC70). HSC70 directly interacts with the AU-rich elements (ARE) of *DICER1* mRNA in both coding and 3′ untranslated region in the presence of MCPyV T-antigen. The T-antigen/HSC70 interaction could induce luciferase activity of synthetic ARE-containing reporter, as well as the stability of ARE-containing mRNAs, suggesting a broader role of MCPyV T-antigens in regulating multiple mRNAs via HSC70. These findings highlight a new role for the interaction of HSC70 and MCPyV T-antigens in mRNA regulation and an undescribed regulatory mechanism of *DICER1* mRNA stability and translation through its direct interaction with HSC70.

## Highlights

• MCPyV T-antigen and HSC70 interaction regulates DICER1 expression
• HSC70 directly binds to ARE in the 3′UTR of *DICER1* for expression regulation
• An unknown motif in *DICER1* CDS is also required for its expression regulation by LT
• The LT-HSC70 interaction can regulate other ARE-containing mRNAs

## Graphical abstract

**Figure:**
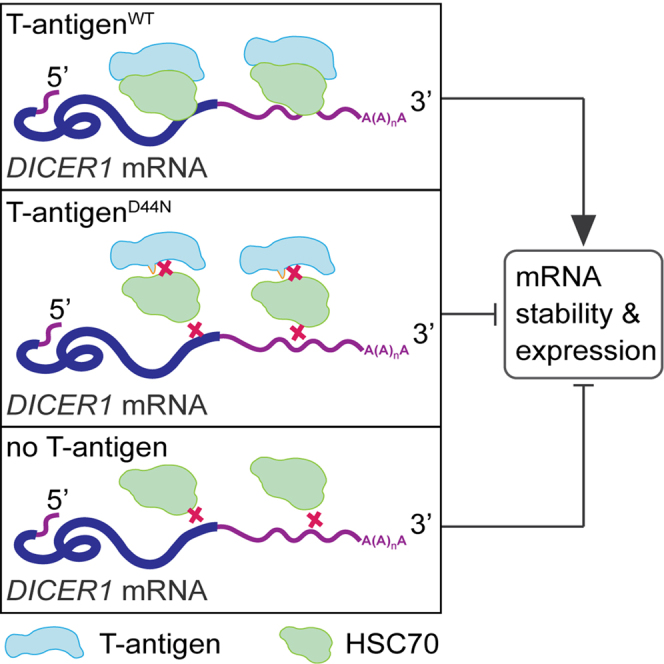

## Introduction

Merkel cell carcinoma (MCC) is a neuroendocrine skin cancer with poor prognosis, of which 80% is caused by Merkel cell polyomavirus (MCPyV) (Feng et al., 2008; Shuda et al., 2008). In MCC, the MCPyV is found integrated into tumor DNA with a mutation in the large T-antigen (LT) gene, expressing truncated large T-antigen (trLT) and small T (sT), which both preserve the DnaJ domain at their N-terminals (Shuda et al., 2008). This domain is conserved among polyomaviruses, which recruits heat shock cognate 70 (HSC70) chaperone (Sullivan and Pipas, 2002). In Simian virus 40 (SV40), this interaction is required for viral replication (Campbell et al., 1997), neoplastic transformation (Srinivasan et al., 1997; Sullivan et al., 2000), and promoting cell-cycle progression (Kim et al., 2001; Sullivan et al., 2000; Zalvide et al., 1998). In MCPyV, a point mutation D44N within this motif disrupts the interaction between HSC70 and MCPyV T-antigen (Kwun et al., 2009), abolishing the effect of the LT on promoting viral replication (Kwun et al., 2009) and cell proliferation (Houben et al., 2015).

HSC70 is a member of the heat shock protein 70 (HSP70) family, which is constitutively expressed and functions as a chaperone that binds nascent polypeptides and facilitates their correct folding for preventing protein aggregation, as well as targets misfolded proteins for degradation (Kim et al., 2013). Besides its protein chaperone functions, HSC70 and various homologs in the HSP70 family can directly bind to selective mRNAs, regulating their stability and translation (Castello et al., 2012; Kishor et al., 2013, 2017; Matsui et al., 2007; Zimmer et al., 2001). RNA binding of HSP70 family members is affected by both ATP binding and co-chaperones (Henics et al., 1999; Zimmer et al., 2001), suggesting that the RNA substrate recognition resembles the protein-binding property of chaperone activity, which may assist in proper folding of its targeted RNA during mRNA decay and/or translation. The underlying mechanism of HSP70 family in mRNA metabolism remains to be uncovered.

HSP70 family proteins preferentially bind to AU-rich elements (ARE), which are *cis*-regulatory elements consisting of AUUUA motif(s) mostly present in the 3′untranslated region (3′UTR) of several mRNAs that typically modulate mRNA decay (Kishor et al., 2013, 2017; Matsui et al., 2007; Zimmer et al., 2001). These chaperone proteins have been suggested to contribute to ARE-mediated decay through two different mechanisms. First, HSP70 proteins may alter the composition of key protein complexes that affect mRNA decay. For example, AU-rich element RNA-binding protein 1 (AUF1), a key ARE-binding protein, binds to poly(A)-binding protein (PABP) and dissociate it from the poly(A) tail, leading to mRNA decay. HSP70 proteins may disrupt the AUF1-PABP interaction through direct HSP70-AUF1 binding that sequesters AUF1 from ARE-mediated mRNA decay; thus PABP remains associated with poly(A) tail and protects the mRNA from ribonucleases (Muhlrad and Parker, 1992). Second, HSP70 may directly bind to ARE and inhibit mRNA decay. For example, Hsc70 binds to ARE in the 3′UTR of *Bim* mRNA and stabilizes the transcript during steady state conditions (Matsui et al., 2007). Upon treatment with interleukin-3, *Bim* mRNA is destabilized (probably through its association with RNA destabilizing factor) with concurrent loss of RNA-binding potential of HSC70 and its interaction with co-chaperones (Matsui et al., 2007). The molecular mechanism of how mRNA stability is regulated by the direct HSC70-RNA interaction is still unclear.

Recently, we observed that T-antigens increased expression of mature microRNAs (miRNA), but not their primary transcripts, through the DnaJ domain of viral T-antigens (Kumar et al., 2020). However, the underlying mechanism of how MCPyV T-antigens regulate miRNA expression remains unknown. One possible mechanism could be the regulation of miRNA biogenesis factors that affect miRNA processing. miRNAs undergo multiple processing steps to produce the functional mature miRNAs (Ha and Kim, 2014). For canonical miRNAs, the primary transcripts are processed by the RNase III enzyme DROSHA together with DiGeorge critical region 8 (DGCR8) in the nucleus, to yield ∼70-nt stem-loop precursor miRNAs. These precursor miRNAs are then exported to the cytoplasm via the Exportin-5/Ran-GTP pathway, for further processing into ∼22-nt miRNA duplexes by another RNase III enzyme DICER1 and its partner transactivation responsive RNA-binding protein 2 (TARBP2). These key proteins are frequently deregulated and associated with disease progression or poor outcomes in multiple tumor types (Merritt et al., 2008; Zhang et al., 2014) and can also contribute to tumorigenesis *in vivo* (Kumar et al., 2007, 2009; Ravi et al., 2012; Zhang et al., 2014). Notably, some viruses have been demonstrated to modulate the expression of miRNA processing factors. For example, human papillomavirus (HPV) oncoproteins can increase DROSHA and DICER1 expressions (Harden and Munger, 2017), whereas Kaposi’s sarcoma-associated herpesvirus (KSHV) can upregulate DICER1 and TARBP2 levels (Happel et al., 2016). These data prompted us to investigate whether MCPyV T-antigens regulate miRNA processing factors.

Here, we report that MCPyV T-antigens induce DICER1 protein expression and mRNA stability through the DnaJ domain and that the interaction between HSC70 and T-antigen is required for DICER1 regulation. In addition, we show that HSC70 directly binds to ARE in the coding region and 3′UTR of *DICER1* mRNA and stabilizes the transcript. These findings reveal a new role for HSC70-binding site of MCPyV T-antigens and the involvement of LT-HSC70 interaction in regulating *DICER1* mRNA stability and protein expression.

## Results

### MCPyV T-antigens induce DICER1 protein expression and stabilize *DICER1* mRNA via DnaJ domain

**Figure 1:**
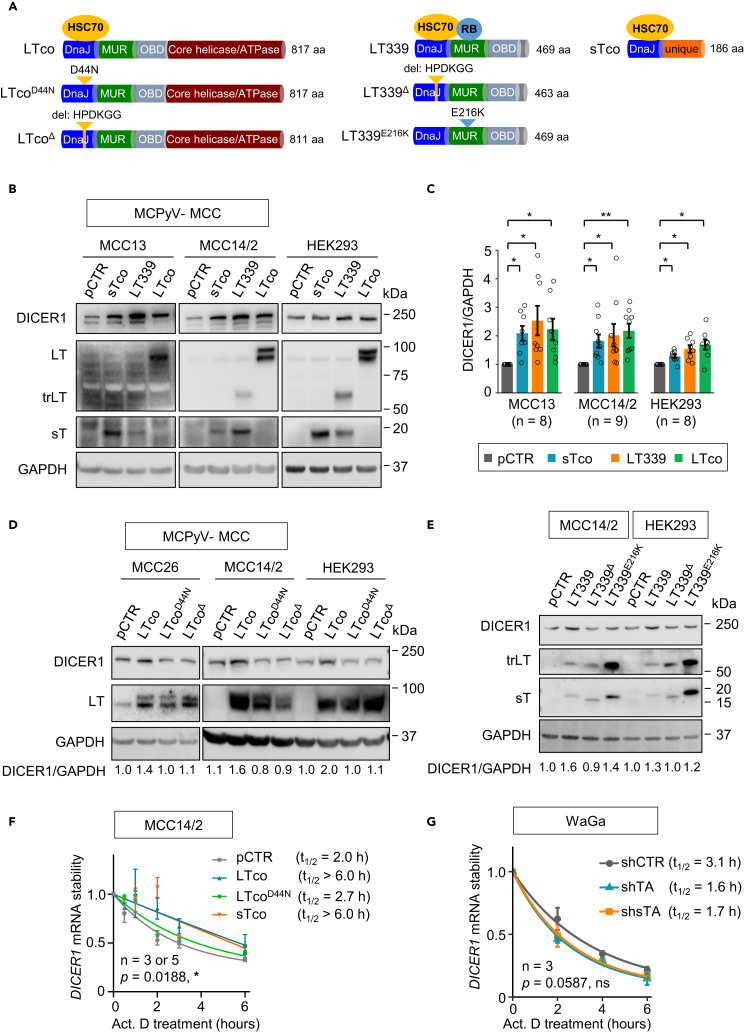
MCPyV T-antigens induce DICER1 protein expression and mRNA stability (A) Illustration of plasmids expressing different MCPyV T-antigens (LTco, full-length LT; LT339, truncated LT (trLT) and sT; sTco, sT only), HSC70-binding defective (LTco^D44N^, LTco^Δ^ and LT339^Δ^), and RB-binding defective (LT339^E216K^) LT mutants. LTco^D44N^ has a substitution of aspartic acid with asparagine at residue 44, whereas both LTco^Δ^ and LT339^Δ^ contain a deletion of the HPDKGG core motif (aa 42-47). LT339^E216K^ has a substitution of glutamic acid with lysine in the LxCxE RB-binding motif. (B) Western blot analysis of the effect of MCPyV T-antigens or vector control (pCTR) on DICER1 expression after 48 h of transfection. trLT, truncated LT. (C) Quantification of DICER1 protein expression in Figure 1B. GAPDH was used for normalization. ∗p < 0.05, ∗∗p < 0.01 by paired t test with Benjamini–Hochberg correction. (D) Western blot analysis of MCPyV− MCC and HEK293 cell lines transfected with LTco, LTco^D44N^, LTco^Δ^, or pCTR after 48 h. (E) Western blots showing the effect of cells transfected with pCTR, LT339, LT339^Δ^, or LT339^E216K^ on DICER1 expression. (F and G) *DICER1* mRNA stability in (F) MCC14/2 cells after transfection with various T-antigens or (G) WaGa cells transfected with shTA, shsTA, or shCTR. Both cell lines were treated with actinomycin D (Act. D, 2 μg/mL) after 48 h. Cells were harvested at 0, 2, 4, and 6 h after Act. D treatment for RT-qPCR analyses. *DICER1* expression was normalized to *GAPDH*, and the treatment at 0 h was set as 1. t_1/2_ refers to the time needed to reach half amount of decaying mRNA. p values were calculated by two-way ANOVA. (B, D, and E) The sizes of the molecular weight markers are given to the right of the western blots. The numbers below the western blots refer to the ratio of DICER1/GAPDH relative to their respective pCTR. (C, F, and G) Each biological replicate is depicted by a circle, or the number of replicates is indicated by n. Error bars represent SEM.

Our previous study has shown that MCPyV T-antigens can induce mature miRNA expressions through DnaJ domain, whereas no changes were observed on corresponding primary miRNAs (Kumar et al., 2020), suggesting that T-antigens can regulate miRNA processing. This finding led us to investigate whether MCPyV T-antigens can regulate key miRNA processing factors. We transfected codon-optimized LT (LTco), tumor-derived truncated LT (LT339), and codon-optimized sT (sTco) into MCPyV− MCC cell lines and evaluated the effect on expression levels of DROSHA, DGCR8, DICER1, and TARBP2 using western blotting (Figures 1A and S1). As compared with DROSHA, DGCR8, and TARBP2, protein expression of DICER1 was markedly increased upon transfection with sTco, LTco, or LT339 (Figure S1). Increased protein expression of DICER1 by MCPyV T-antigens was consistently observed in multiple cell lines (Figures 1B and 1C).

Given that the DnaJ domain of MCPyV T-antigens is required for regulation of miRNA expression (Kumar et al., 2020), we determined whether the HSC70-binding site in the DnaJ domain of MCPyV T-antigens is required for DICER1 expression regulation. To address this question, we constructed two types of HSC70-binding defective mutants, LTco^D44N^ and LTco^Δ^. The LTco^D44N^ mutant has a substitution of aspartic acid with asparagine at residue 44, whereas the LTco^Δ^ contains a deletion of the HPDKGG (aa 42-47) core motif of DnaJ domain (Figure 1A). These mutants exhibited loss of interaction between HSC70 and LT, as evaluated by co-immunoprecipitation (co-IP) (Figures S2A and S2B). Indeed, we observed that both mutants abolished the effect of LT on increased DICER1 protein expression (Figure 1D).

To further support our findings, we also generated the HPDKGG deletion mutant in LT339 (LT339^Δ^; Figures 1A and S2C) and a point substitution in the LxCxE motif (LT339^E216K^). The E216K mutation has been reported to abolish RB binding to LT (Shuda et al., 2008), and the defective RB binding to LT339 was verified by co-IP (Figure S2D). Unlike LTco, LT339 expressed both trLT and sT (Figure 1E). Concordant with LTco, the LT339^Δ^ DnaJ mutant failed to induce DICER1 protein expression, whereas increased DICER1 expression was still observed in the RB-binding defective LT339^E216K^ mutant (Figure 1E).

In addition, both LT and sT prolonged *DICER1* mRNA half-life (t_1/2_ > 6 h) compared with vector control (pCTR) (t_1/2_ = 2.0 h), whereas LTco^D44N^ had subtle effect (t_1/2_ = 2.7 h), as assessed by inhibition of transcription using actinomycin D treatment, followed by RT-qPCR (Figure 1F). Similarly, silencing of both LT and sT by shTA, or sT alone by shsT, decreased mRNA stability of *DICER1* (Figure 1G). These findings suggest that MCPyV T-antigens enhance DICER1 protein expression and mRNA stability and that the DnaJ domain is required for this regulation.

### MCPyV T-antigen is required for HSC70-mediated DICER1 regulation

**Figure 2:**
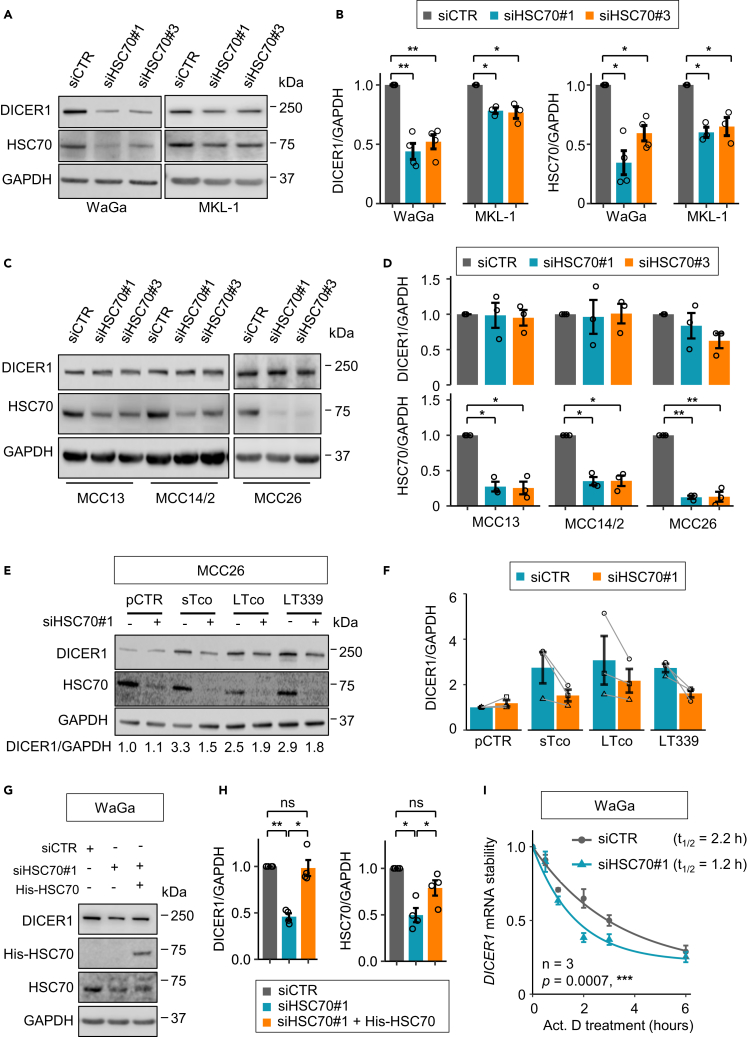
HSC70 is required for MCPyV T-antigen-mediated regulation of DICER1 protein expression and mRNA stability (A) MCPyV+ cell lines were transfected with two different siRNAs targeting HSC70 (siHSC70#1 and siHSC70#3) or siRNA control (siCTR) for 48 h, followed by evaluation of the effect on DICER1 expression using western blotting. (B) Quantification of DICER1 and HSC70 protein expressions in Figure 2A. (C) Western blot analysis of MCPyV− MCC cell lines transfected with siHSC70 or siCTR after 48 h of transfection. (D) Quantification of DICER1 and HSC70 in Figure 2C. (E) MCC26 cells were co-transfected with various T-antigens and siHSC70#1 (+) or siCTR (−). The numbers below the western blots refer to the DICER1/GAPDH ratios relative to cells transfected with pCTR and siCTR. (F) Quantification of DICER1/GAPDH in Figure 2E. The paired data are connected by gray lines. Error bars, SEM. (G) Western blot analysis of the effect of ectopically expressed siRNA-resistant His-tagged HSC70 (His-HSC70) on DICER1 expression in WaGa cells transfected with siHSC70 or siCTR. (H) Quantification of DICER/GAPDH in Figure 2G. (I) *DICER1* mRNA stability in WaGa cells transfected with siCTR or siHSC70#1. Actinomycin D (Act. D, 2 μg/mL) was added after 48 h of transfection, followed by RT-qPCR. *GADPH* was used as endogenous control. *∗∗∗*p < 0.001 by two-way ANOVA. (B, D, and H) GAPDH was used as loading control. Expression levels were normalized to siCTR. Error bars, SEM. ∗p < 0.05, ∗∗p < 0.01, ∗∗∗p < 0.001 and ns, not significant by paired t test with Benjamini–Hochberg correction. Each biological replicate is depicted by a circle.

To further investigate whether HSC70 is required for regulating DICER1 expression, we silenced HSC70 using two different siRNAs (siHSC70#1 and siHSC70#3) in MCPyV+ WaGa and MKL-1 cell lines. Indeed, silencing of HSC70 resulted in decreased expression of DICER1 (Figures 2A and 2B). However, no significant change of DICER1 protein expression was observed in MCPyV− MCC cell lines upon silencing of HSC70 (Figures 2C and 2D), indicating that HSC70 only regulates DICER1 expression in the presence of viral T-antigen. To support this notion, we compared the effect of T-antigens on DICER1 with and without silencing of HSC70. DICER1 expression was increased (2.5–3.3 folds) upon ectopic expressions of T-antigen (sTco, LTco or LT339) compared with pCTR; however, HSC70 knockdown partially diminished the effect (Figures 2E and 2F).

To further strengthen the involvement of HSC70 in MCPyV T-antigen-mediated DICER1 regulation, we assessed whether ectopically expressed siRNA-resistant His-tagged HSC70 (His-HSC70) could recue the silencing effect of HSC70 in MCPyV+ WaGa cells. The results showed that expression of His-HSC70 reversed the effect of HSC70 knockdown on DICER1 expression (Figures 2G and 2H). In addition, inhibition of HSC70 in the MCPyV+ WaGa cells reduced *DICER1* mRNA stability (Figure 2I). Together, our results indicate that MCPyV T-antigens regulate DICER1 protein expression and mRNA stability through HSC70 and that the interaction between MCPyV T-antigen and HSC70 is crucial for DICER1 regulation.

### HSC70 directly binds to 3′UTR and CDS of *DICER1*

**Figure 3:**
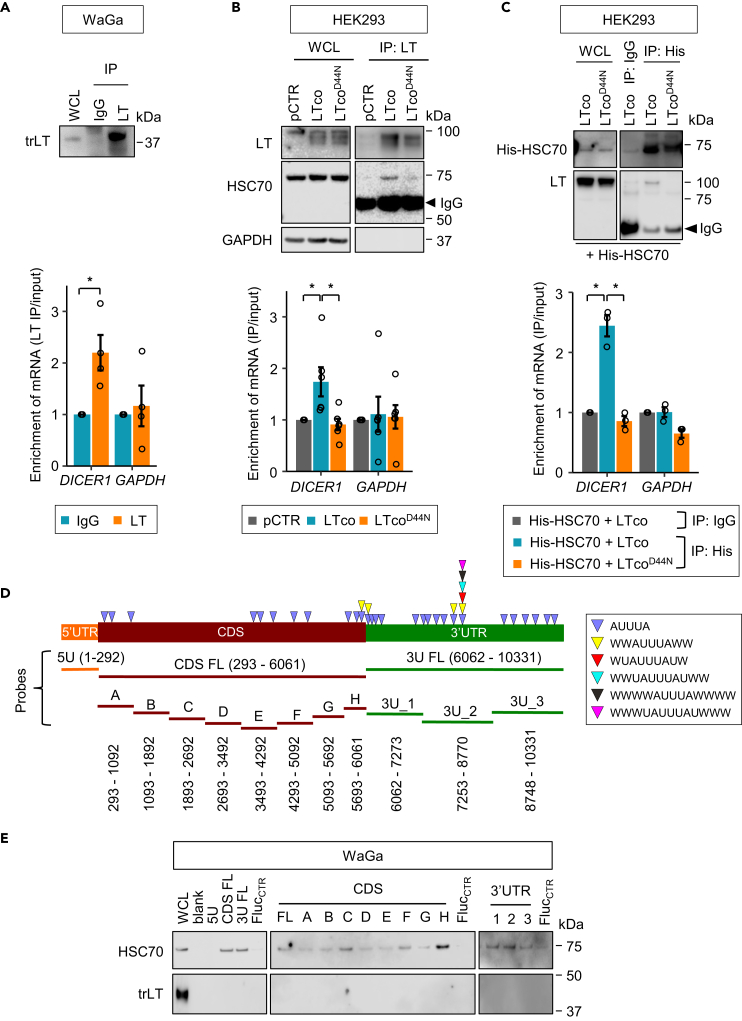
*DICER1* mRNA is present in the LT-HSC70 complex and directly associated with HSC70 (A–C) Evaluation of *DICER1* mRNA in LT or His-HSC70 immunoprecipitation (IP). RNA immmunoprecipitation was performed on (A) WaGa cells or (B) HEK293 cells transfected with LTco, LTco^D44N^ or pCTR, using LT antibody, as well as on (C) HEK293 cells co-transfected with His-HSC70 together with LTco or LTco^D44N^ using His-tagged antibody. *Upper*: IP efficiency was validated by western blotting. IgG IP or pCTR was used as a negative control. WCL, whole-cell lysate. *Lower*: quantification of *DICER1* and *GAPDH* mRNA in LT or His IP using RT-qPCR. Enrichment of mRNA was calculated by dividing the mRNA in LT IP to input and compared with their expression levels in IgG IP control (A and C) or pCTR (B). Error bars, SEM. ∗p < 0.05 by paired t test. Each biological replicate is depicted as a circle. (D) Illustration of *DICER1* mRNA and various RNA probes synthesized for biotin pull-down assays. Probes spanning the full length of 5′UTR, CDS, and 3‘UTR of *DICER1* are indicated by 5U (orange), CDS FL (red), and 3U FL (green), respectively. Fragment probes spanning the CDS are presented as (A) to (H) (red), whereas the probes for 3’UTR fragments are shown as 3U_1, 3U_2, and 3U_3 (green). The numbers next to the probe name refer to the nucleotide positions in the transcript (ENST00000526495.5). Arrowheads indicate various ARE motifs predicted by AREsite (Gruber et al., 2010). (E) Biotinylated RNA probes (20 pmol) were incubated with protein lysates from WaGa cells for 5–6 h at 4°C, and then pulled down by streptavidin beads. The proteins bound to *DICER1* RNA probes were detected by western blotting. Biotinylated RNA probes from firefly luciferase gene (Fluc_CTR_) were included in each experiment as a negative control. WCL, whole-cell lysate; blank, empty lane. The same blots with high and low exposure for HSC70 detection are shown in Figure S3A.

Given the involvement of LT-HSC70 interaction in regulation of DICER1 mRNA stability and protein expression, we assessed whether this interaction binds directly to *DICER1* mRNA. We first pulled down RNAs bound to LT using RNA immunoprecipitation (RIP) and quantified *DICER1* mRNA by RT-qPCR. The results showed an enrichment of *DICER1*, but not *GAPDH*, mRNA in LT immunoprecipitates compared with IgG control (Figure 3A), suggesting that *DICER1* mRNA is a part of LT-HSC70 ribonucleoprotein complex. To further determine the importance of LT-HSC70 interaction on *DICER1* mRNA binding, we performed RIP on HEK293 cells transfected with pCTR, LTco, or LTco^D44N^ and found that *DICER1* mRNA was significantly enriched in LTco-, but not LTco^D44N^, transfected cells compared with pCTR (Figure 3B). We also made several attempts to pull down endogenous HSC70 in MCPyV+ MCC cell lines using different HSC70 antibodies (sc-7298 from Santa Cruz Biotechnology, NBP2-67335 from Novus Biologicals and ab51052 from Abcam). Unfortunately, none of these antibodies could pull down the HSC70 complexes (data not shown), which is likely due to lack of accessible epitopes for the antibodies. Instead, we ectopically expressed His-HSC70 in HEK293 cells and pulled down exogenous HSC70 using His-tagged antibody (Figure 3C). The results showed an enrichment of *DICER1* mRNA in cells co-transfected with His-HSC70 together with LTco but not with LTco^D44N^ (Figure 3C).

We next investigated the interaction between LT-HSC70 and *DICER1* mRNA using biotin pull-down assays with biotinylated RNA probes from full-length 5′ UTR (5U), CDS (CDS FL), and 3′UTR (3U FL) of *DICER1* (Figures 3D and 3E). We detected HSC70 in the ribonucleoprotein complex using probes spanning *DICER1* CDS and 3′UTR but not its 5′UTR or negative control (Fluc_CTR_, biotinylated RNA probe from firefly luciferase gene). However, LT was not detected in the complex, suggesting that *DICER1* is directly associated with HSC70 but not with LT.

To further map specific regions of *DICER1* that binds to HSC70, we generated 8 probes spanning the CDS and 3 probes spanning the 3′UTR of *DICER1* (Figure 3D). Among the CDS probes, CDS_H (nt 5693–6061) showed the strongest association with HSC70 (Figures 3E and S3A). Furthermore, the 3U_2 (nt 7253–8770) in 3′UTR of *DICER1* also showed strong interaction with HSC70 (Figures 3E and S3A). Interestingly, both regions contain nonameric motifs of ARE (WWAUUUAWW; Figure 3D), suggesting that HSC70 may preferentially bind to these motifs of *DICER1*.

To further define the role of T-antigen in HSC70-*DICER1* mRNA interaction, we repeated the biotin pull-down assays using the CDS_H and 3U_2 probes in MCPyV+ (WaGa and MKL-1) and the MCPyV− MCC14/2 cell lines. Consistently, HSC70 was detected in the ribonucleoprotein complex from both MCPyV+ cell lines, but barely detectable in the MCPyV− MCC14/2 cell line (Figure S3B), indicating that T-antigen is required for the HSC70-*DICER1* mRNA interaction. Overall, our results indicate that HSC70 directly binds to the CDS and 3′UTR of *DICER1* mRNA in MCPyV+ MCC cell lines.

### The ARE motif in the *DICER1* 3′UTR is important for MCPyV T-antigen-mediated expression regulation

**Figure 4:**
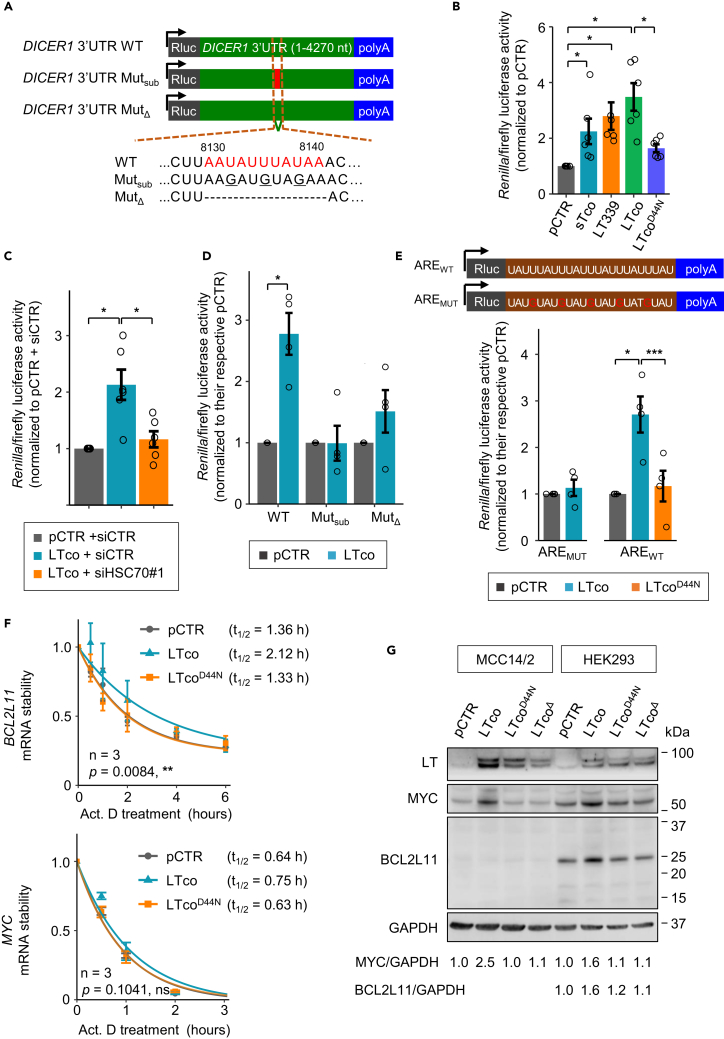
ARE motif in the 3′UTR contributes to gene expression regulation by MCPyV T-antigen (A) Illustration of various *Renilla* luciferase reporters containing wild-type (WT) or mutations (Mut) of *DICER1* 3′UTR. Two types of mutants were generated: Mut_sub_ contains three nucleotide substitutions (underlined), and Mut_Δ_ has a deletion of the ARE motif (nt 8130–8140), which are highlighted in red. (B and C) Luciferase activity was assessed in HEK293 cells after 48 h of co-transfection with *DICER1* 3′UTR WT luciferase reporter and (B) various MCPyV T-antigens or (C) pCTR or LTco together with siHSC70 or siCTR. (D) The effect of LTco on luciferase activity from reporters containing wild-type (WT) or mutations (Mut_sub_ and Mut_Δ_) of *DICER1* 3′UTR was evaluated in HEK293 cells after 48 h of transfection. (E) *Upper*: illustration of *Renilla* luciferase reporters containing synthetic ARE sequences (ARE_WT_) or ARE mutant (ARE_MUT_). In the ARE_MUT_, three U > G substitutions were introduced in the middle U of the pentameric ARE motif. *Lower*: the graph shows the effect of LTco and LTco^D44N^ on ARE_WT_ luciferase activity, as well as the effect of LTco on ARE_MUT_ luciferase activity in HEK293 cells. (B–E) Error bars, SEM; ∗p < 005, ∗∗∗p < 0.001 by paired t test with Benjamini–Hochberg correction for multiple comparisons. Each biological replicate is depicted as a circle. (F) RT-qPCR analysis of *BCL2L11* and *MYC* in HEK293 cells transfected with pCTR, LTco, or LTco^D44N^ after addition of actinomycin D (Act. D) at the indicated time points. mRNA levels were normalized to GAPDH. p values were calculated by two-way ANOVA. (G) Western blot analysis of MYC and BCL2L11 in cells transfected with pCTR, LTco, and HSC70-binding defective LT mutants (LTco^D44N^ and LTco^Δ^). MYC/GAPDH and BCL2L11/GAPDH ratios relative to pCTR are shown below the western blots.

To determine the role of *DICER1* 3′UTR in expression regulation, we co-transfected *Renilla* luciferase reporter containing the wild-type sequence of *DICER1* 3′UTR and various MCPyV T-antigens into HEK293 cells (Figures 4A and 4B). Our results showed that all T-antigens enhanced luciferase activity compared with vector control (pCTR). On the other hand, LTco^D44N^ or silencing of HSC70 abolished the effect mediated by LTco (Figures 4B and 4C).

To dissect the role of ARE in this regulation, we selected an ARE (5′-AAUAUUUAUAA-3′ nt 8130–8140; Figure S4), located within the 3U_2 region, which showed strong association with HSC70 by biotin pull-down assay (Figure 3E). We generated two mutant constructs, where either three U > G substitutions were created (Mut_sub_) or the whole ARE motif was deleted (Mut_Δ_) (Figure 4A). Consistently, LTco enhanced luciferase activity of *DICER1* 3′UTR WT, but not the luciferase activity of *DICER1* 3′UTR Mut_sub_ and Mut_Δ_ (Figure 4D), suggesting that this ARE in *DICER1* 3′UTR is important for T-antigen regulation of DICER1 expression. In addition, RNAfold (http://rna.tbi.univie.ac.at//cgi-bin/RNAWebSuite/RNAfold.cgi) predicted a linear secondary structure of this ARE region, making it accessible for HSC70 binding (Figure S4).

To determine whether MCPyV T-antigen functions in general ARE-mediated gene regulation, we cloned an artificial ARE sequence (ARE_WT_) or mutant ARE (ARE_MUT_) into *Renilla* luciferase vector and evaluated the effect of LTco or LTco^D44N^ on these reporter activities (Figure 4E). Similar to the observations for *DICER1* 3′UTR reporter, LTco, but not LTco^D44N^, induced luciferase activity of the ARE_WT_, and no enhanced luciferase activity was observed in the ARE_MUT_ (Figure 4E). These findings suggest that LT-HSC70 interaction can regulate multiple ARE-containing mRNAs. To address this question, we quantified mRNA half-life of two genes, *BCL2L11* and *MYC*, which have *cis*-regulatory ARE within their mRNA (Brewer and Ross, 1988; Matsui et al., 2007). The mRNA half-life of *BCL2L11* was increased by LTco, whereas LTco^D44N^ showed no effect on its mRNA stability (Figure 4F). Concordantly, the BCL2L11 protein level was also induced by LTco but not by the HSC70-binding defective LT mutants (Figure 4G). Although LTco had a subtle effect on *MYC* mRNA stability (Figure 4F), its protein level was markedly induced in cells transfected with LTco compared with pCTR or the HSC70-binding defective LT mutants (Figure 4G). These data suggest that MCPyV T-antigens can regulate a number of mRNAs containing ARE via HSC70.

### Deletion of nonameric ARE in the CDS of *DICER1* partly reverses the effect of LT

**Figure 5:**
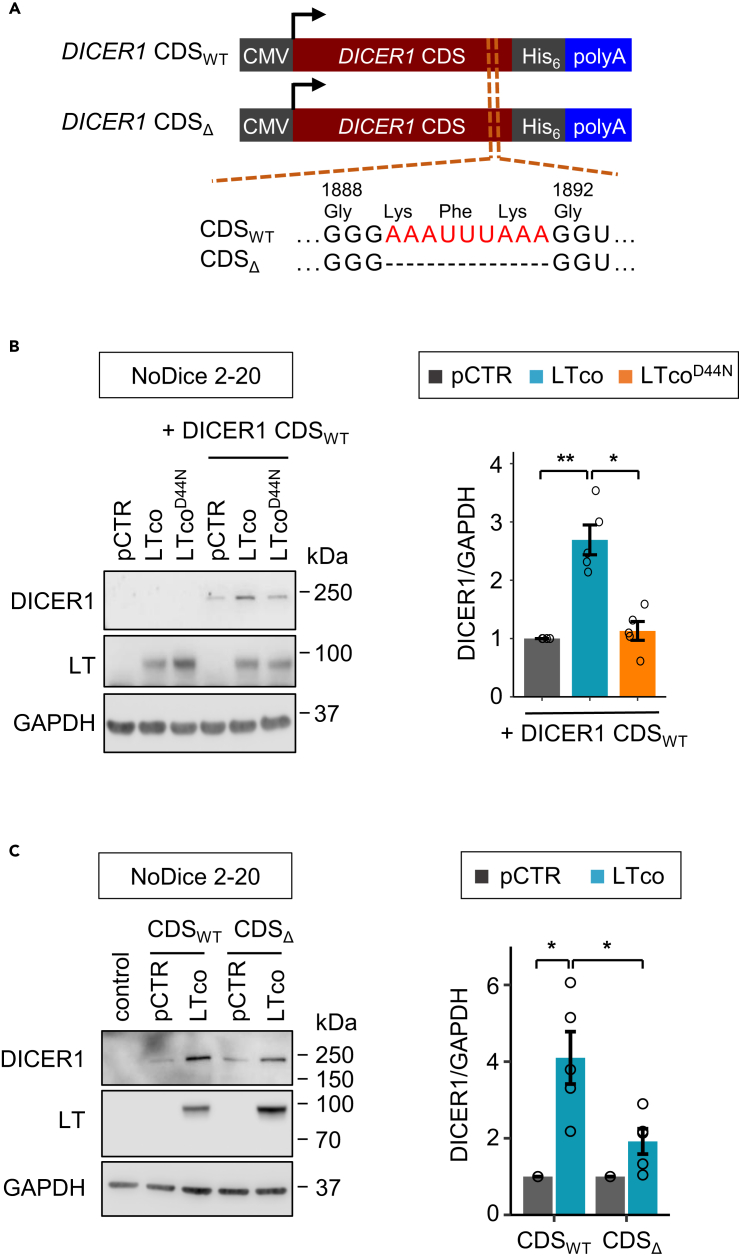
The nonameric ARE in the CDS partly contributes to LT-induced DICER1 expression (A) Illustration of plasmids containing the full-length CDS of *DICER1* (CDS_WT_) or an in-frame deletion of aa1889-1891, which corresponds to the nonameric ARE (CDS_Δ_). (B) Western blot analysis of NoDice 2-20 cells co-transfected with *DICER1* CDS_WT_ and LTco, LTco^D44N^, or pCTR. (C) The effect of LTco on exogenous DICER1 expression from *DICER1* CDS_WT_ and CDS_Δ_ was evaluated in NoDice 2-20 cells using western blotting. (B and C) *Left*: representative western blots showing the transfection efficiency and the effect on DICER1 expression. GAPDH was used as loading control; control, untransfected NoDice 2-20 cells. *Right*: quantification of DICER1 expression after normalization to GAPDH and pCTR was set to 1. Error bars, SEM. ∗p < 0.05, ∗∗p < 0.01 by paired t test with Benjamini–Hochberg correction for multiple comparisons. Each biological replicate is depicted by a circle.

Our biotin pull-down assays revealed that HSC70 interacted with the CDS of *DICER1* and fragment H (nt 5693–6061) had the strongest interaction (Figure 3E). We determined whether *DICER1* CDS alone (without 3′UTR) is sufficient to regulate DICER1 expression, by co-transfecting a plasmid expressing the full-length CDS of *DICER1* together with T-antigen or pCTR in *DICER1* knockout cell line (NoDice 2-20) (Figures 5A and S5A). Our results showed that all T-antigens (sTco, LTco and LT339) increased expression of exogenous DICER1 (Figure S5A). However, the HSC70-binding defective LT mutant (LTco^D44N^) or HSC70 knockdown (siHSC70#1) abolished the effect of T-antigen-induced DICER1 expression (Figures 5B and S5B).

To assess whether the nonameric ARE in the fragment H of *DICER1* CDS can regulate DICER1 expression, we constructed an in-frame deletion mutant, which has a deletion of the nonameric ARE in the CDS of *DICER1* (CDS_Δ_; Figure 5A). By comparing the effect of LT in NoDice 2-20 cells, we observed 4-fold increased expression of DICER1 in the CDS_WT_, and < 2-fold in CDS_Δ_ (Figure 5C), suggesting that the nonameric ARE in the *DICER1* CDS partly contributes to expression regulation by the viral T-antigens.

### AUF1 does not contribute to DICER1 expression regulation in MCC

**Figure 6:**
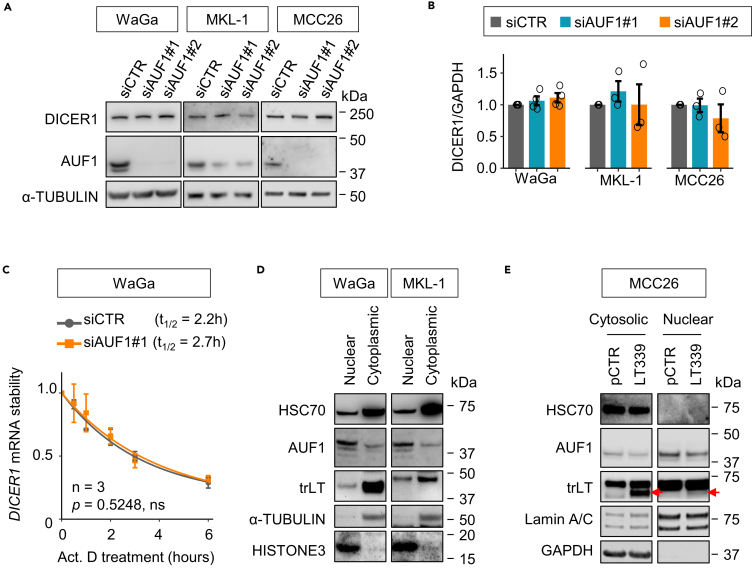
AUF1 is not involved in DICER1 regulation in MCC (A) Western blot analysis of DICER1 expression upon AUF1 silencing in MCPyV+ (WaGa and MKL1) and MCPyV− (MCC26) cell lines. Two different siRNAs targeting AUF1 (siAUF1#1 and siAUF#2) were used. siCTR was applied as a negative control. (B) Quantification of DICER1 expression in Figure 6A. DICER1 expression was normalized to GAPDH and then siCTR. Error bars, SEM. Each biological replicate is presented as a circle. (C) RT-qPCR analysis of *DICER1* expression in WaGa cells transfected with siCTR or siAUF1 after actinomycin D (Act. D) treatment. This experiment was conducted in parallel to the HSC70 silencing experiments described in Figure 2I. p value was assessed by two-way ANOVA. ns, not significant. (D) Western blot analysis of HSC70 and AUF1 in nuclear and cytoplasmic fractions of MCPyV+ cell lines. α-TUBULIN and HISTONE3 were used as cytoplasmic and nuclear markers, respectively. (E) Western blot analysis of HSC70 and AUF1 in cytosolic and nuclear compartments of MCC26 cells transfected with LT339 or pCTR. Lamin A/C was used as nuclear or nuclear membrane marker, whereas GAPDH was used as cytoplasmic marker. Red arrows indicate trLT of LT339.

To our knowledge, AUF1 is the only RNA-binding protein that binds to *DICER1* mRNA and destabilize the transcripts (Abdelmohsen et al., 2012). To determine whether AUF1 plays a role in DICER1 regulation in MCC, we silenced AUF1 using two different siRNAs (siAUF1#1 and siAUF1#2) in MCPyV+ (WaGa and MKL-1) and MCPyV− (MCC26) cell lines and evaluated the effect on DICER1 protein expression. Silencing of AUF1 did not alter DICER1 protein expression in any of the three cell lines (Figures 6A and 6B). Similarly, no changes of *DICER1* mRNA stability were observed upon silencing of AUF1 in WaGa (Figure 6C). In addition, no AUF1 was detected in the *DICER1* biotin pull-down assays (Figures S3A and S3B). Furthermore, protein fractionation experiments revealed that AUF1 was predominantly present in the nuclear fraction, whereas HSC70 and endogenous/exogenous LT were highly abundant in the cytoplasm (Figures 6D and 6E), suggesting that LT-HSC70 interaction binds to *DICER1* mRNA in the cytoplasm, thus enhancing its mRNA stability and translation. Our results also indicate that AUF1 is not involved in DICER1 regulation in MCC.

## Discussion

This study reveals a novel role for LT-HSC70 interaction in RNA regulation. Particularly, we show that this interaction regulates *DICER1* mRNA stability and protein expression through the ARE located in the CDS and 3′UTR of *DICER1*.

### MCPyV T-antigens posttranscriptionally regulate DICER1 expression via HSC70

Here, we demonstrate that (1) ectopic expression of MCPyV T-antigen or silencing of endogenous T-antigens affect *DICER1* mRNA stability; (2) *DICER1* mRNA is enriched in the LT-HSC70 ribonucleoprotein complex but not in the complex of HSC70-binding defective LT mutant; (3) *DICER1* mRNA directly interacts with HSC70. In addition, we also observed that ectopic expression of MCPyV T-antigens did not affect proteolysis of DICER1 (data not shown), excluding the possibility that the increased expression of DICER1 protein level was due to inhibition of protein degradation pathway.

It is noteworthy that both sT and LT can regulate DICER1 expression through HSC70. Although we did not generate HSC70-binding defective sTco^D44N^, the HPDKGG deletion mutant of LT339 (LT339^Δ^) expressed both trLT and sT. Notably, both trLT and sT were detected in His IP from cells expressing LT339 but not LT339^Δ^ (Figure S2C). These results support that sT also binds to HSC70 and the deletion of the HPDKGG motif abolished its interaction with HSC70. In addition, ectopic expression of sTco also induced DICER1 protein expression, and silencing of HSC70 reversed the effect of sTco (Figure 2E). Collectively, these data support that both LT and sT regulate DICER1 expression via HSC70.

The identification of *DICER1* mRNA as a direct target of HSC70 in the present study also expands the involvement of HSC70 in regulation of mRNA stability in a broader context. Prior to this study, Hsc70 was only known to bind to *Bim* mRNA in a cytokine-dependent manner (Matsui et al., 2007). It is noted that cochaperones, Bag-4, CHIP, Hsp40, and Hip, are required for Hsc70 binding to *Bim* mRNA (Matsui et al., 2007). Similarly, MCPyV T-antigen is required for HSC70-*DICER1* mRNA interaction and DICER1 expression regulation; thus, the DnaJ domain of MCPyV T-antigen can act as cochaperone for regulating the RNA-binding potential of HSC70. Similar to *Bim* (Matsui et al., 2007), we show that AUF1 is not involved in regulating mRNA stability of *DICER1*. These findings suggest that HSC70-mediated mRNA stabilization of *Bim* and *DICER1* is unlikely due to disruption of AUF1-PABP interaction, leading to sequestration of AUF1 from its mRNA decay activity. Alternatively, both data support direct interaction between HSC70 and mRNAs that contributes to mRNA stability via an unknown mechanism.

### ARE in the 3′UTR of *DICER1* contributes to expression regulation by the LT-HSC70 interaction

Here, we demonstrate that HSC70 directly binds to CDS and 3′UTR of *DICER1*, and both regions of *DICER1* can contribute to expression regulation by the LT-HSC70 interaction. Similarly, AUF1 has also been shown to interact with both CDS and 3′UTR of *DICER1* mRNA (Abdelmohsen et al., 2012). These findings suggest that sequence elements residing within these regions can affect *DICER1* expression. At the posttranscriptional level, the 3′UTR is the most common site for expression regulation. This region usually contains regulatory elements for mRNA decay, such as ARE- and miRNA-binding sites (Mayr, 2017). For *DICER1*, multiple miRNAs, e.g. *miR-103*/*107* family (Martello et al., 2010), *miR-192* (Feinberg-Gorenshtein et al., 2013), and *miR-630* (Rupaimoole et al., 2016), have been demonstrated to target its 3′UTR. Besides miRNAs, to our knowledge, only two ARE-binding proteins, AUF1 and HSC70, are known to interact with the 3′UTR of *DICER1*. Notably, the HSC70 interaction sites of *DICER1* 3′UTR are similar to AUF1, in which only the first half of the 3′UTR interacts with both HSC70 and AUF1. Although it is unclear whether HSC70 and AUF1 bind to the same ARE site(s) of *DICER1* 3′UTR, we could not detect AUF1 in our biotin pull-down assays in both MCPyV+ and MCPyV− MCC cell lines. Furthermore, silencing of AUF1 did not affect DICER1 protein expression and mRNA stability. These findings suggest that HSC70 is unlikely competing with AUF1 for the same ARE sites, releasing *DICER1* from AUF1-mediated mRNA decay. Whether the direct interaction between HSC70 and mRNAs affect mRNA stability through inhibition of deadenylation has yet to be uncovered. Alternatively, the binding of HSC70 to *DICER1* 3′UTR may inhibit miRNAs from associating with their target sites, thereby releasing *DICER1* from miRNA-mediated repression. Similar mechanism has also been observed for other RNA-binding proteins, such as Dnd1 (Kedde et al., 2007) and RBM38 (Léveillé et al., 2011), in which both proteins bind to uridine-rich regions near miRNA target sites and block miRNA accessibility.

### A coding region determinant in *DICER1* mRNA is required for LT-mediated DICER1 expression

Although ARE are mostly described within the 3′UTR of mRNAs, mapping of ARE-binding proteins’ binding sites using high-throughput method, e.g. PAR-CLIP, has identified binding motifs beyond 3′UTR, including CDS (e.g. LARP4B [Küspert et al., 2015; Van Nostrand et al., 2020]) and intronic regions (e.g. HuR [Lebedeva et al., 2011; Mukherjee et al., 2011]). We therefore investigated fragment H of the *DICER1* CDS (nt 5693–6061), which showed the strongest interaction with HSC70 and has a putative nonameric ARE. Although deletion of the ARE significantly reduced the effect of T-antigen compared with *DICER1* CDS_WT_, LT still increased DICER1 expression with CDS_Δ_, but to a lesser extent. These results suggest that other sequences in fragment H or other regions within the *DICER1* CDS may also contribute to *DICER1* expression regulation. Nevertheless, this motif still partly contributes to increased DICER1 expression by T-antigen.

HSP70 family proteins, including HSC70, are associated with polysomes, and these chaperones have been demonstrated to enhance translation elongation by regulating nascent peptide folding, leading to continued elongation (Liu et al., 2013; Shalgi et al., 2013). In addition, HSP70 proteins can also enhance translation initiation by maintaining interaction with Pab1, the yeast homolog of human PABP (Horton et al., 2001; Zhong and Arndt, 1993), or solubility of eIF4G (Cuesta et al., 2000), which strengthens the translation initiation complexes. In both scenarios, HSP70/HSC70 is not expected to bind directly to mRNAs. However, the LT-HSC70 interaction could still recruit translational complexes for mRNA translations.

Alternatively, *DICER1* mRNA may contain a translation-dependent coding region determinant (CRD) that regulates its expression. It is known that some mRNAs contain CRD that can affect their stability through endonucleolytic cleavage or deadenylation during translation (Bernstein et al., 1992; Grosset et al., 2000; Mattijssen et al., 2017; Noubissi et al., 2006; Wisdom and Lee, 1991). There are several examples, such as *c-myc* (Bernstein et al., 1992; Wisdom and Lee, 1991), *c-fos* (Grosset et al., 2000), *β-TrCP* (Noubissi et al., 2006), and *LARP4* (Mattijssen et al., 2017). For c-*myc*, the CRD is located in the last 249 nucleotides of the CDS, which can trigger rapid mRNA turnover (Wisdom and Lee, 1991). This region is associated with an RNA-binding protein, called CRD-BP (also known as IGF2BP1), which protects the CRD from degradation by polysome-associated endonuclease(s) during translational pausing (Lemm and Ross, 2002). Given that translational pausing is linked to heat shock (Shalgi et al., 2013) and proteotoxic stress (Liu et al., 2013), it is tempting to speculate that the interaction between HSC70 and *DICER1* CDS may protect the mRNA from endonucleolytic attack, deadenylation, and/or enhance mRNA translation.

### LT-HSC70-mediated RNA regulation and potential clinical implications

In addition to DICER1, we also show that the LT-HSC70 interaction can induce luciferase activity of synthetic ARE-containing reporter, as well as mRNA stability or protein expression of endogenous ARE-containing mRNAs, *BCL2L11*, and *MYC*. These results suggest that the LT-HSC70 interaction can regulate multiple mRNAs. Further investigations are warranted to map the LT-mediated HSC70-mRNA interactions on a genome-wide scale and investigate the functional roles of these interactions in MCC.

Although the clinical implications of our findings are presently unknown, HSC70 is the most abundantly expressed HSP70 family member in MCC cell lines (Adam et al., 2014). Importantly, MAL3-101, an inhibitor of HSP70 ATPase activity, has been demonstrated to induce apoptosis in MCC cell lines and inhibit tumor growth in MCC xenograft model (Adam et al., 2014), suggesting that inhibition of HSP70/HSC70 activity can be a potential therapeutic strategy for MCC treatment.

Based on our findings, we propose a model that the DnaJ domain of T-antigen recruits HSC70 that binds to *DICER1* mRNA in both CDS and 3′UTR, leading to mRNA stabilization and enhanced protein expression. In HSC70-binding defective mutant of T-antigen or without MCPyV T-antigen, HSC70 can no longer bind to *DICER1* mRNA and regulate its expression.

### Limitations of study

This study demonstrates that MCPyV T-antigens, including both LT and sT, regulate DICER1 mRNA stability and protein expression through HSC70. Although the enrichment of *DICER1* mRNA in His-HSC70 immunoprecipitates of cells expressing LT and detection of HSC70 using *DICER1* mRNA probes in the biotin pull-down assays provide strong evidence of direct HSC70-*DICER1* mRNA interaction, we were unable to pull down endogenous HSC70 using several commercial HSC70 antibodies. The evidence of direct interaction with the endogenous HSC70 will further establish the significance of our findings.

Another limitation of this study is the underlying mechanism of how the HSC70-*DICER1* mRNA interaction contributes to *DICER1* mRNA stability and translation. Further experiments are warranted to investigate whether distinct mechanisms are involved for HSC70-mediated regulation of *DICER1* 3′UTR and CDS. Although the functional roles of MCPyV T-antigen-mediated DICER1 regulation have not been investigated in this study, we previously demonstrated that the DnaJ domain of MCPyV LT is required for miRNA regulation (Kumar et al., 2020). However, it is still unclear how the viral T-antigens contribute to selective miRNA processing. One possible explanation could be attributed to the binding of RNA-binding proteins, e.g. KSRP (Trabucchi et al., 2009) and lin28 (Heo et al., 2009; Viswanathan et al., 2008), which can promote or prevent processing of their targeted precursor miRNAs.

Although our current data indicate that the LT-HSC70 interaction can regulate multiple ARE-containing mRNAs, other LT-HSC70-regulated mRNAs have yet to be identified using high-throughput sequencing approach and functionally characterized. The results of these studies will shed new insights into the mechanisms and functions of MCPyV T-antigens in viral infection and tumorigenesis.

## STAR★Methods

### Key resources table

**Table:** 

REAGENT or RESOURCE	SOURCE	IDENTIFIER
**Antibodies**		

Rabbit polyclonal anti-AUF1 (HNRNPD) antibody	Novus Biologicals	NBP1-88915 RRID: AB_11037375
Mouse monoclonal anti-BCL2L11 antibody (H-5)	Santa Cruz Biotechnology	sc-374358 RRID: AB_10987853
Rabbit monoclonal anti-DGCR8 antibody (EPR18757)	Abcam	ab191875 RRID: AB_2892625
Rabbit monoclonal anti-DICER1 antibody (D38 × 10^7^)	Cell Signaling Technology	5362 RRID: AB_10692484
Mouse monoclonal anti-DROSHA antibody (C-7)	Santa Cruz Biotechnology	sc-393591 RRID: AB_2732793
Rabbit monoclonal anti-GADPH antibody (14C10)	Cell Signaling Technology	5174 RRID: AB_10622025
Mouse monoclonal anti-His-Tag antibody (H-3)	Santa Cruz Biotechnology	sc-8036 RRID: AB_627727
Mouse monoclonal anti-Histone H3 antibody (1G1)	Santa Cruz Biotechnology	sc-517576 RRID: AB_2848194
Mouse monoclonal anti-HSC70 antibody (B-6)	Santa Cruz Biotechnology	sc-7298 RRID: AB_627761
Rabbit monoclonal anti-HSC70 antibody (SR39-04)	Novus Biologicals	NBP2-67335 RRID: NBP2-67335
Mouse monoclonal anti-MCPyV large T-antigen antibody (CM2B4)	Santa Cruz Biotechnology	sc-136172 RRID: AB_2013156
Mouse monoclonal anti-MCPyV T-antigen antibody (clone 2t2)	Merck KGaA	MABF2316 RRID: MABF2316
Mouse monoclonal anti-MYC antibody (9E10)	Santa Cruz Biotechnology	sc-40 RRID: AB_2857941
Rabbit polyclonal anti-RB1 antibody (C-15)	Santa Cruz Biotechnology	sc-50 RRID: AB_632339
Mouse monoclonal anti-TARBP2 (TRBP2) antibody (D-5)	Santa Cruz Biotechnology	sc-514124 RRID: AB_2732792
Mouse monoclonal anti-α-TUBULIN antibody (B-7)	Santa Cruz Biotechnology	sc-5286 RRID: AB_628411
IRDye 680RD Goat anti-Rabbit IgG Secondary Antibody	LI-COR Biosciences	926-68071 RRID: AB_10956166
IRDye 800CW Goat anti-Mouse IgG Secondary Antibody	LI-COR Biosciences	926-32210 RRID: AB_621842

**Bacterial and virus strains**		

TOP10 Chemically Competent E. coli	Thermo Fisher Scientific	C404010

**Chemicals, peptides, and recombinant proteins**		

TRIzol Reagent	Thermo Fisher Scientific	15596026
Actinomycin D	Sigma-Aldrich	A1410
Lipofectamine LTX Reagent with PLUS Reagent	Thermo Fisher Scientific	15338030
Lipofectamine™ 3000 Transfection Reagent	Thermo Fisher Scientific	L3000008
Ingenio electroporation solution	Mirus Bio LLC	MIR50111
Pierce BCA Protein Assay Kit	Thermo Fisher Scientific	23225
SuperSignal West Femto Maximum Sensitivity Substrate	Thermo Fisher Scientific	34095
Protein G Sepharose 4 Fast Flow beads	Sigma-Aldrich	GE17-0618-01
Dynabeads M-280 Streptavidin	Thermo Fisher Scientific	11205D
Biotinylated UTP (Bio-11-UTP)	Thermo Fisher Scientific	AM8450
LDS loading buffer	Thermo Fisher Scientific	NP0008
Sample Reducing Agent	Thermo Fisher Scientific	NP0009
RIPA buffer	Thermo Fisher Scientific	89900
NP40 buffer	Thermo Fisher Scientific	FNN0021
RNaseOUT Recombinant Ribonuclease Inhibitor	Thermo Fisher Scientific	10777019
Phenylmethanesulfonyl fluoride	Sigma-Aldrich	93482
Dithiothreitol	Thermo Fisher Scientific	P2325
Proteinase inhibitor	Sigma-Aldrich	P8340
MultiScribe Reverse Transcriptase	Thermo Fisher Scientific	4311235
Platinum Taq DNA Polymerase	Thermo Fisher Scientific	10966018

**Critical commercial assays**		

TaqMan gene expression assay: *DICER1*	Thermo Fisher Scientific	Hs00229023_m1
TaqMan gene expression assay: *GAPDH*	Thermo Fisher Scientific	Hs99999905_m1
TaqMan gene expression assay: BCL2L11	Thermo Fisher Scientific	Hs01076940_m1
TaqMan gene expression assay: MYC	Thermo Fisher Scientific	Hs00153408_m1
Monarch PCR & DNA Cleanup Kit	New England Biolabs	T1030
HiScribe T7 High Yield RNA Synthesis Kit	New England Biolabs	E2040S
MEGAclear Transcription Clean-Up Kit	Thermo Fisher Scientific	AM1908
Dual-Glo Luciferase Assay	Promega Biotech	E2920
Q5 Site-Directed Mutagenesis Kit	New England Biolabs	E0554
Qproteome Cell Compartment Kit	Qiagen	37502

**Experimental models: Cell lines**		

MCPyV- MCC13	CellBank Australia	CBA-1338 RRID: CVCL_2583
MCPyV- MCC14/2	CellBank Australia	CBA-1340 RRID: CVCL_ 2584
MCPyV- MCC26	CellBank Australia	CBA-1341 RRID: CVCL_2585
MCPyV+ WaGa	Laboratory of Jürgen Becker	RRID: VCL_E998
MCPyV+ MKL-1	Laboratory of Nancy Krett	RRID: CVCL_2600
HEK293	ATCC	Cat# CRL-1573 RRID: CVCL_0045
NoDice 2-20	Bogerd et al. (2014)	N/A

**Oligonucleotides**		

*In vitro* transcription of DICER1 RNA probes (5' to 3')		
5′UTR forward: TAATACGACTCACTATAGGGGCGGAAGTGGGTGTTTGTTA	This paper	N/A
5′UTR reverse: TCATCCAGTGTTTCTTTCATTGC	This paper	N/A
CDS_A forward: TAATACGACTCACTATAGGGATGAAAAGCCCTGCTTTGCA	This paper	N/A
CDS_A reverse: TAAAGCCCACTTCTGTCAGT	This paper	N/A
CDS_B forward: TAATACGACTCACTATAGGGTGAAAGACTGCTGATGGAAT	This paper	N/A
CDS_B reverse: CCAAGTTGCATTTTGGTATA	This paper	N/A
CDS_C forward: TAATACGACTCACTATAGGGTGGTTCGTTTTGATTTGCCCA	This paper	N/A
CDS_C reverse: TCTTGTGGTATCTTCAGGAG	This paper	N/A
CDS_D forward: TAATACGACTCACTATAGGTGCTTTGGAATACTGACGGC	This paper	N/A
CDS_D reverse: GCAGTCAAAAGGCAGTGAAG	This paper	N/A
CDS_E forward: TAATACGACTCACTATAGGAGAGGAGCTAAGAGCCCAGA	This paper	N/A
CDS_E reverse: TGCAAAATAGATATGTGGTG	This paper	N/A
CDS_F forward: TAATACGACTCACTATAGGCTTACCCTGATGCGCATGAG	This paper	N/A
CDS_F reverse: TTCCCGATCAGTCCTTTTAA	This paper	N/A
CDS_G forward: TAATACGACTCACTATAGGAAGGCCCTGTGCCCTACTCG	This paper	N/A
CDS_G reverse: CTCTTCTTTCTCTTCATCCT	This paper	N/A
CDS_H forward: TAATACGACTCACTATAGGGATATTGAAGTTCCAAAGGC	This paper	N/A
CDS_H reverse: TCAGCTATTGGGAACCTGAG	This paper	N/A
3U_1 forward: TAATACGACTCACTATAGGGTCACTACTAGTGTTCT AGAAACCGC	This paper	N/A
3U_1 reverse: ACGGCAGTTTATCGCAAACGT	This paper	N/A
3U_2 forward: TAATACGACTCACTATAGGGACGTTTGCGATAAA CTGCCGT	This paper	N/A
3U_2 reverse: CAGTCCTTTACACACGTGCTCAG	This paper	N/A
3U_3 forward: TAATACGACTCACTATAGGGCTGAGCACGTGTG TAAAGGACTG	This paper	N/A
3U_3 reverse: CCGCGAACAGACGATAACTTTAT	This paper	N/A
Site-directed mutagenesis:		
LTco^Δ^ sense: AACCCAGTCATTATGATG	This paper	N/A
LTco^Δ^ antisense: GTGTTTCAAACAGGATCG	This paper	N/A
LT339^Δ^ sense: AATCCTGTTATAATGATGGAATTG	This paper	N/A
LT339^Δ^ antisense: ATGCTTTAAGCAGCTTCTTTTG	This paper	N/A
LT339^E216K^ sense: CTTCTGCGATAAATCACTTTCCTCCC	This paper	N/A
LT339^E216K^ antisense: AGATCCTCCCAGGTGCCA	This paper	N/A
*DICER1* 3′UTR Mut_sub_ sense: TAGAAACTACCTGATGCCAGGA	This paper	N/A
*DICER1* 3′UTR Mut_sub_ antisense: CATCTTAAGAGATTTGTGATTAAACTTGATC	This paper	N/A
*DICER1* 3′UTR Mut_Δ_ sense: ACTACCTGATGCCAGGAG	This paper	N/A
*DICER1* 3′UTR Mut_Δ_ antisense: AAGAGATTTGTGATTAAACTTGATCAC	This paper	N/A
*DICER1* CDS_Δ_ sense: AGGGGAAATTCAAAGGTGTTG	This paper	N/A
*DICER1* CDS_Δ_ antisense: TTCCTACTACTTCCACAG	This paper	N/A
Artificial ARE constructs		
ARE_MUT_ sense: CTAGTTATGTATGTATGTATGTATGTATGC	This paper	N/A
ARE_MUT_ antisense: GGCCGCATACATACATACATACATACATAA	This paper	N/A
ARE_WT_ sense: CTAGTTATTTATTTATTTATTTATTTATGC	This paper	N/A
ARE_WT_ antisense: GGCCGCATAAATAAATAAATAAATAAATAA	This paper	N/A
siRNA (uppercase, DNA; lowercase, RNA)		
siAUF1#2 sense: acgaggaggaugaaggccaTT	Raineri et al. (2004)	N/A
siAUF1#2 antisense: uggccuucauccuccucguTT	Raineri et al. (2004)	N/A
siHSC70#1	Qiagen	SI02661477
siHSC70#3	Qiagen	SI04236596
siAUF1#1	Qiagen	SI00300454

**Recombinant DNA**		

LTco (pcDNA6 MCV LTco)	Shuda et al. (2011)	Addgene Cat#40200 RRID: Addgene_40200
LTco^Δ^	This paper	N/A
LTco^D44N^	Kwun et al. (2009)	N/A
sTco (pcDNA6 MCV sTco)	Shuda et al. (2011)	Addgene Cat #40201 RRID: Addgene_40201
LT339 (pcDNA6 MCV LT339 V5.4)	Shuda et al. (2008)	Addgene Cat#28193 RRID: Addgene_28193
LT339^Δ^	This paper	N/A
LT339^E216K^	This paper	N/A
His-HSC70 (pcDNA5/FRT/TO HIS HSPA8)	Hageman and Kampinga (2009)	Addgene Cat#19541 RRID: Addgene_19541
shTA (shRNA targeting LT and sT)	Xie et al. (2014)	N/A
shsT (shRNA targeting sT)	Xie et al. (2014)	N/A
DICER1 3′UTR (pIS1 DICER1 long UTR)	Mayr and Bartel (2009)	Addgene Cat#21649 RRID: Addgene_21649
DICER1 3′UTR Mut_sub_	This paper	N/A
DICER1 3′UTR Mut_Δ_	This paper	N/A
ARE_MUT_	This paper	N/A
ARE_WT_	This paper	N/A
DICER1 CDS_WT_	Paulsson et al. (2020)	N/A
DICER1 CDS_Δ_	This paper	N/A

**Software and algorithms**		

R version 4.0.5	https://www.R-project.org/	N/A; RRID:SCR_001905
Rstudio	http://www.rstudio.com/	N/A; RRID:SCR_000432
Rstatix	https://github.com/kassambara/rstatix	N/A; RRID:SCR_021240
tidyverse	https://www.tidyverse.org/	N/A; RRID:SCR_019186
Prism GraphPad 8.0	GraphPad Software, La Jolla California USA	N/A; RRID:SCR_002798

### Resource availability

#### Lead contact

Further information and requests for reagents should be directed to the lead contact, Weng-Onn Lui (weng-onn.lui@ki.se).

#### Materials availability

Plasmids generated in this study are available upon request.

### Experimental model and subject details

Cell lines used in this study include human MCC cell lines [WaGa (male), MKL-1 (male), MCC13 (female), MCC14/2 (male) and MCC26 (female)], human embryonic kidney cell line HEK293 (female) and *DICER1* knockout cell line NoDICE 2-20 (derived from HEK293T). All MCC cell lines were verified by short tandem repeat profiling as previously reported (Kumar et al., 2020). Loss of DICER1 expression was verified in NoDice 2-20 cells by Western blotting. Refer to the key resources table for information on the source of each cell line and to the method details below for culture conditions.

### Method details

#### Cell culture

MCC cell lines were cultured in RPMI1640 medium supplemented with 10% (MCPyV+: WaGa and MKL-1) or 15% (MCPyV-: MCC13, MCC14/2 and MCC26) fetal bovine serum (FBS). HEK293 and NoDice 2-20 cell lines were cultured in DMEM media with 1 g/L glucose (HEK293) or 4.5 g/L glucose (NoDice 2-20) supplemented with 10% FBS.

#### Plasmids and siRNAs

MCPyV codon-optimized full-length LT (LTco; Addgene, #40200), truncated LT (LT339; Addgene, #28193), HSC70 expression vector (His-HSC70 contains the full-length coding sequence of *HSPA8*, NM_006597; Addgene, #19541), *DICER1* 3′UTR luciferase reporter (*DICER1* 3′UTR; Addgene, #21649) and firefly luciferase reporter (Addgene, #64784) plasmids were purchased from Addgene (Cambridge, MA, USA). Codon-optimized sT (sTco) and LTco^D44N^ were kindly provided by Drs. Yuan Chang and Patrick Moore (University of Pittsburgh, Pennsylvania, USA). The vector control for viral T-antigen (pCTR) was previously constructed by deleting the insert from the LT339 plasmid (Xie et al., 2014). The *DICER1* CDS_WT_ contains the full-length coding sequence (CDS) of *DICER1* inserted between SalI and NotI restriction sites of pCMV6entry expression vector, which had been described in our previous study (Paulsson et al., 2020). MCPyV T-antigen mutant constructs (LTco^Δ^, LT339^Δ^ and LT339^E216K^) and *DICER1* 3′UTR mutant constructs (Mut_sub_, Mut_Δ_ and CDS_Δ_) were generated with the Q5 Site-Directed Mutagenesis Kit (New England Biolabs, Ipswich, MA, USA; #E0554). Primers used for mutagenesis are listed in key resources table. For artificial ARE-containing luciferase reporter constructs, synthetic oligonucleotides containing wild-type (WT) or mutated (MUT) sequences of the ARE were phosphorylated, annealed and cloned into *DICER1* 3′UTR luciferase reporter backbone after digestion with SpeI and NotI (New England Biolabs, Ipswich, MA, USA). All mutant and ARE-containing vectors were confirmed by Sanger sequencing at the KIGene core facility.

Short hairpin RNA (shRNA) plasmids targeting either both MCPyV LT and sT (shTA), or only sT (shsT) were previously generated in our laboratory (Xie et al., 2014). Small interfering RNAs (siRNAs) silencing *HSPA8*/*HSC70* (siHSC70#1, SI02661477; siHSC70#3, SI04236596), *AUF1* (siAUF1#1, SI00300454) and siRNA control (siCTR, #1027310) were purchased from Qiagen (Hilden, Germany). SiAUF1#2, was designed in (Raineri et al., 2004) and oligos were synthesized by Integrated DNA Technologies (Leuven, Belgium). The oligos were phosphorylated using T4 polynucleotide kinase (New England Biolabs) and annealed in 1X annealing buffer (50 mM Tris, pH 7.5–8.0; 100 mM NaCl). All oligonucleotides used for plasmid constructions and siRNAs are detailed in Key resources table.

#### Transfection

MCPyV+ cell lines (WaGa and MKL-1) were transfected using Nucleofector electroporation system. Briefly, 3 million cells were pelleted and re-suspended in 100 μL Ingenio electroporation solution (MIR50111; Mirus Bio LLC, Madison, WI, USA) containing 2 μg plasmid DNA or 30 pmol siRNA. The mixture was transferred to a cuvette and electroporated using program D-24 (WaGa) or A-23 (MKL-1) of the Nucleofector™ 2b Device (Lonza, Basel, Switzerland). MCPyV- cell lines (MCC14/2, MCC26 and MCC13) were transfected with 2–10 μg plasmid DNA or 20 pmol siRNA using Lipofectamine LTX with PLUS (Thermo Fisher Scientific, #15338100). Similar procedures were applied to HEK293 and NoDice 2-20 cell lines, where Lipofectamine 3000 transfection reagent (Thermo Fisher Scientific, #L3000150) was used.

#### Western blot analysis

Whole cell lysates were prepared using RIPA buffer (Thermo Fisher Scientific, #89900) supplemented with protease inhibitor (Sigma-Aldrich, #P2714) and 1% phenylmethanesulfonyl fluoride (Sigma-Aldrich, #93482). Protein concentrations were assessed using Pierce BCA Protein Assay Kit (Thermo Fisher Scientific, #23225). Protein lysates (30 μg) were separated in 4–12% NuPAGE Bis-Tris gels, and transferred to nitrocellulose membranes (Thermo Fisher Scientific, #LC2000). After blocking with 5% skim milk, the membranes were incubated with primary antibodies at 4°C overnight. After washing, the membranes were incubated with HRP- or fluorescence-conjugated antibodies for 1 h at room temperature. For HRP-conjugated secondary antibodies, detection was performed using SuperSignal West Femto Maximum Sensitivity Substrate (Thermo Fisher Scientific, #34095). Images were acquired using Odyssey Fc Image system (LI-COR Biosciences, Lincoln, NE, USA) and analyzed in Image Studio Lite (LI-COR Biosciences, Lincoln, NE, USA). Antibodies used for Western blotting are detailed in key resources table.

#### RNA stability analysis

Actinomycin D (2 μg/mL, Sigma-Aldrich, #A1410) was added to the cells after 48 h of transfection, and cells were harvested at indicated time points after treatment. Cells were washed with cold PBS twice, and total RNA was isolated using Trizol reagent (Thermo Fisher Scientific, #15596018). RNA concentrations were measured using a Nanodrop Spectrophotometer (Thermo Fisher Scientific). Complementary DNA (cDNA) was synthesized with MultiScribe Reverse Transcriptase (Thermo Fisher Scientific, #4311235). TaqMan gene expression assays for *DICER1* (Hs00229023_m1), *GAPDH* (Hs99999905_m1), *BCL2L11* (Hs01076940_m1) and *MYC* (Hs00153408_m1) were purchased from Thermo Fisher Scientific (Waltham, MA). Relative mRNA expressions were first normalized to *GAPDH* using 2^−ΔCT^, and then compared to time point 0.

#### Immunoprecipitation (IP) and RNA-IP (RIP)

To prepare antibody-beads conjugation, 150 μL beads slurry (Protein G Sepharose 4 Fast Flow beads, GE17-0618-01, Sigma-Aldrich) was washed three times with 1 mL NT2 buffer (50 mM Tris-HCl, pH 7.4; 150 mM NaCl; 1 mM MgCl2; 0.5% NP-40) supplemented with 8 U/mL RNase inhibitor (RNaseOUT; Thermo Fisher Scientific; #10777019), followed by incubation with anti-His (Santa Cruz Biotechnology, sc-8036, 1:50 dilution), anti-HSC70 (Santa Cruz Biotechnology, sc-8036, 1:50 dilution), anti-HSC70 (Novus Biologicals, NBP2-67335, 1:50 dilution), anti-HSC70 (Abcam, ab51052, 1:100 dilution) or anti-LT (Santa Cruz Biotechnology, sc-136172, 1:50 dilution) for 5–6 hours at 4°C. Unbound antibodies were washed away in NT2 buffer.

Forty-eight hours after transfection, cells were washed with cold PBS twice and lysed in NP40 buffer (Thermo Fisher Scientific, FNN0021), supplemented with 200 U/mL RNase inhibitor, 1% phenylmethanesulfonyl fluoride (Sigma-Aldrich, #93482), 1 mM Dithiothreitol (Thermo Fisher Scientific, P2325) and proteinase inhibitor (Sigma-Aldrich, P8340).

To pull down RNAs bound to target proteins, 3 mg of whole cell lysate was added to the antibody-beads slurry, supplemented with EDTA (1 M), RNase inhibitor (200 U/mL) and protease inhibitor. After incubation overnight at 4°C on a rotator, sepharose beads were pelleted and washed 4 × 10 minutes with 1 mL cold NT2 buffer, supplemented with RNase inhibitor 8 U/mL. For Western blot analysis, beads were eluted with LDS loading buffer (Thermo Fisher Scientific, NP0008) and Sample Reducing Agent (Thermo Fisher Scientific, NP0009), and the lysates were denatured and then subjected to 4-12% Bis-Tris gel electrophoresis. For RNA analysis, proteinase K (1 mg/mL) was added to beads slurry, and then incubated at 55°C for 30 minutes. Total RNA was isolated with Trizol Reagent and RT-qPCR was performed to quantify the mRNA levels of *DICER1* and *GAPDH*. Enrichment of mRNA was calculated by dividing the amount of mRNAs in the IP samples to their input samples.

#### *In vitro* transcription and biotin pull-down assay

To prepare DNA templates for *in vitro* transcription (IVT), *DICER1* fragments were amplified using Platinum Taq DNA Polymerase (Thermo Fisher Scientific, #10966018) with primers detailed in key resources table. *DICER1* CDS and *DICER1* 3′UTR plasmids were used as templates for amplifying coding sequence and 3′UTR fragments, respectively. Total cDNA of WaGa cells was used as template for amplifying the 5′UTR of *DICER1*. PCR products were purified with the Monarch PCR & DNA Cleanup Kit (New England Biolabs, #T1030), and were then used for IVT with the HiScribe T7 High Yield RNA Synthesis Kit (New England Biolabs, #E2040S). Probes were labeled with biotinylated UTP (Bio-11-UTP, Thermo Fisher Scientific, #AM8450), cleaned by MEGAclear Transcription Clean-Up Kit (Thermo Fisher Scientific, #AM1908) and quantified using a Nanodrop Spectrophotometer (Thermo Fisher Scientific).

For pull-down assay, 20 pmol of biotinylated RNA probe was incubated with 300 μg of whole cell lysate for 5–6 hours at 4°C. Protein complexes were isolated with 40 μL of Dynabeads M-280 Streptavidin (Thermo Fisher Scientific, #11205D). After washing twice with 300 μL washing buffer (5 mM Tris-HCl, pH 7.5; 0.5 mM EDTA; 1 M NaCl) for 2 min, the pull-down mixture was analyzed by Western blotting. A biotinylated RNA probe from firefly luciferase gene (Fluc_CTR_) was used as a control for non-specific binding.

#### Luciferase reporter assay

One μg of plasmid DNA encoding *Renilla* luciferase with various 3′UTR constructs (*DICER1* 3′UTR WT, *DICER1* 3′UTR Mut_sub_, *DICER1* 3′UTR Mut_Δ,_ ARE_WT_ or ARE_MUT_) together with 1 μg of firefly luciferase reporter plasmid and 1 μg of T-antigen plasmid (sTco, LT339, LTco, LTco^D44N^ or pCTR) or 20 pmol siHSC70#1/siCTR were co-transfected into 2×10^5^ HEK293 cells using Lipofactamine 3000. Luciferase activity was assessed using Dual-Glo Luciferase Assay (Promega Biotech, #E2920) and luminescence was detected by Spark 10M multimode microplate reader (Tecan Group Ltd, Männedorf, Switzerland). All experiments were performed in triplicates for each condition and repeated at least three times independently. The *Renilla* luciferase activity was divided by the firefly luciferase activity in each condition and normalized to the pCTR or siCTR.

#### Subcellular fractionation

MCPyV+ cells, WaGa and MKL-1 (∼1×10^7^), were collected and washed with cold PBS twice. 200 μL subcellular fractionation buffer (20 mM HEPES, pH 7.4; 10 mM KCl; 2mM MgCl_2_; 1mM EDTA; 2mM DTT; supplemented with protease inhibitor cocktail) were added to cell pellet and ground 10 times using a pestle. After 20-minute incubation on ice, the lysate was centrifuged at 700 g for 5 min and the supernatant was transferred to a new tube as cytoplasmic fraction. The remaining nuclear pellet was washed with subcellular fractionation buffer twice, and then resuspended in 200 μL RIPA buffer. After 20-minute incubation on ice, the nuclei were disrupted by sonication 3 × 15s in ice-cold water, followed by centrifugation at 16,000 g for 30 minutes to collect the supernatant as nuclear fraction. MCPyV- MCC26 cells were collected after 48 hours of transfection and fractionated using Qproteome Cell Compartment Kit (Qiagen, Cat#37502). Protein concentration was assayed using Pierce BCA Protein Assay Kit (Thermo Fisher Scientific, #23225), and ∼50 μg of total protein was loaded for Western blot analysis. α-TUBULIN or GAPDH was used as a cytoplasm marker, while HISTONE3 or Lamin A/C was used as a nuclear marker.

### Quantification and statistical analysis

Statistical tests were performed in R (version 4.0.5, Windows 10) using the package “rstatix”, unless specified otherwise. Two-tailed paired t-test was used to compare two groups in transfection assays. p-values for multiple tests were corrected with “Benjamini–Hochberg” method. RNA stability assay was analyzed in GraphPad Prism 8 (GraphPad Software, Inc, CA, USA), and one-phase decay model was applied to determine the half-life of mRNAs (t_1/2_). p-values for mRNA stability analysis were calculated by two-way ANOVA. Statistical significance was defined as a p < 0.05 and p values were indicated by asterisks as ∗p < 0.05, ∗∗p < 0.01, ∗∗∗p < 0.001 and ns, not significant. Error bars stand for standard error of the mean (SEM), and numbers of observations or replicates (n) are indicated in figure legends.

## Data Availability

• All source data for Figures 1C, 1F, 1G, 2B, 2D, 2F, 2H, 2I, 3A–3C, 4B–4F, 5B, 5C, 6B, 6C, S1, and S5A, as well as uncropped Western blot images were deposited in Mendeley data (https://doi.org/10.17632/r3gphxxs3b.1). • There is no original code reported in this work. • Any additional information required is available from the lead contact upon reasonable request.
