# Supplementary material for: Merkel cell polyomavirus T-antigens regulate DICER1 mRNA stability and translation through HSC70
Source: iScience. 2021 Oct 14;24(11):103264. doi: 10.1016/j.isci.2021.103264 (PMC8567380; doi:10.1016/j.isci.2021.103264)
Supplement: Document S1. Figures S1–S5 [file mmc1.pdf]

iScience, Volume *24*

Supplemental information

Merkel cell polyomavirus T-antigens regulate *DICER1* mRNA stability and translation through HSC70

Jiwei Gao, Hao Shi, C Christofer Juhlin, Catharina Larsson, and Weng-Onn Lui

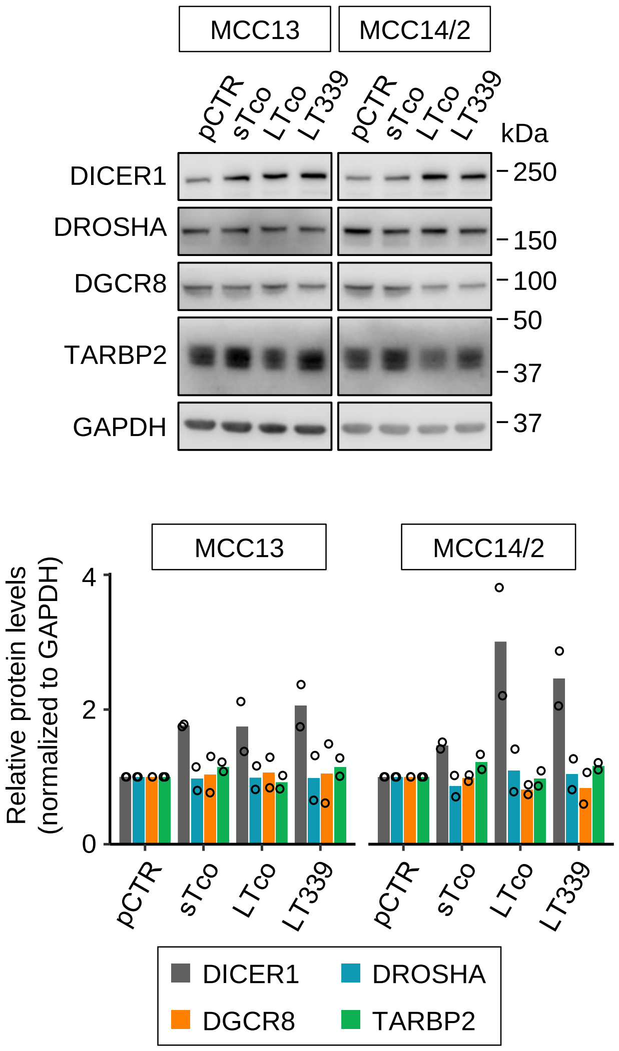

Figure S1. Effect of MCPyV T-antigens on miRNA key processing factors, related to Figure 1.
Upper: Representative Western blots showing the effect of various T-antigens on DICER1, DROSHA, DGCR8 and TARBP2 expressions. GAPDH was used as loading control.
Lower: Quantification of miRNA processing proteins upon overexpression of MCPyV T-antigen. Protein levels were normalized to GAPDH. Relative protein levels of each protein were compared to pCTR. Data represent mean of two independent replicates.

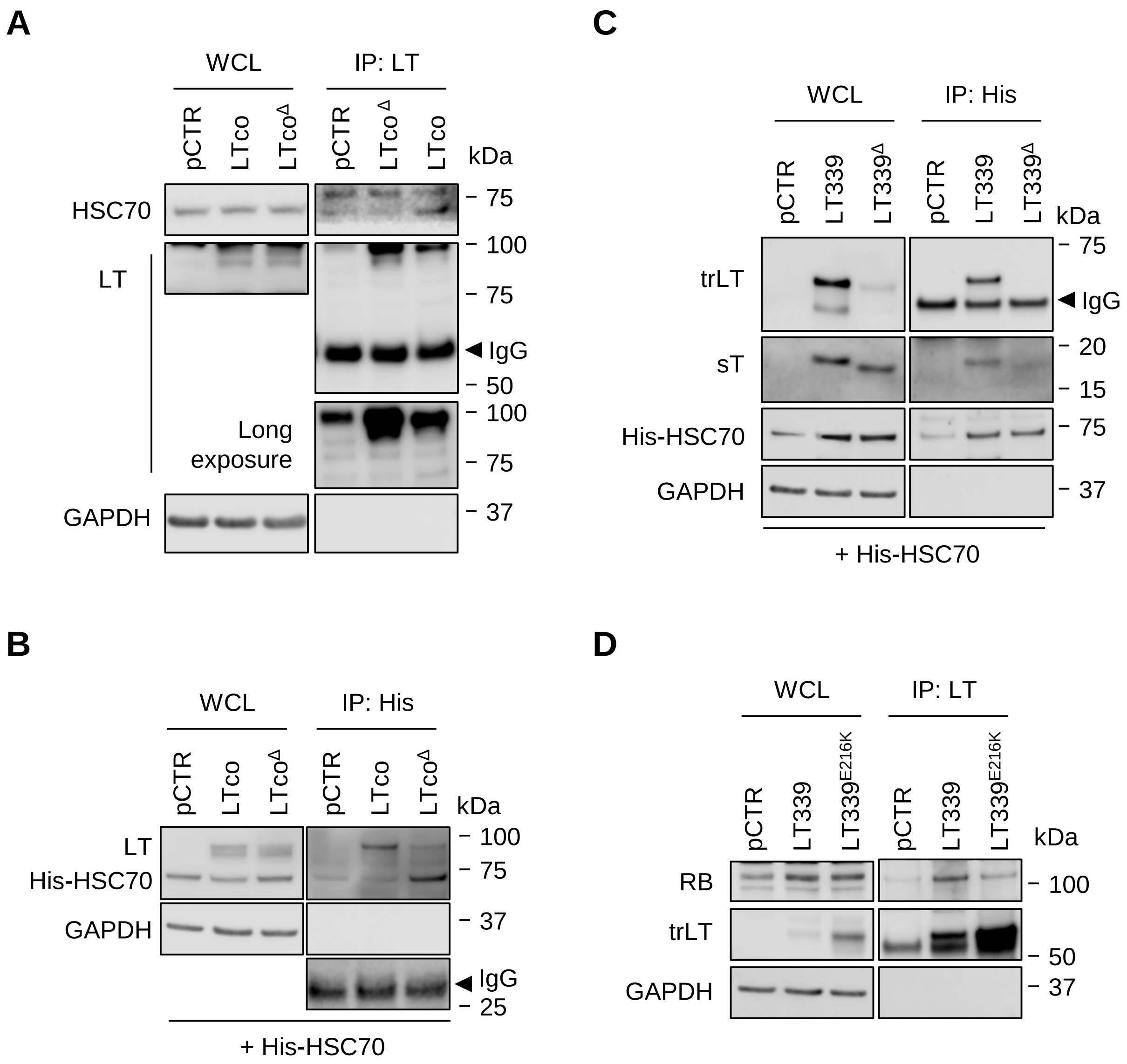

Figure S2. Validations of defective HSC70 or RB binding in the LTco$^{\Delta}$, LT339$^{\Delta}$ and LT339^{E216K} mutants in HEK293 cells by co-immunoprecipitation (co-IP), related to Figure 1.

(A) Detection of endogenous HSC70 in LT IP from cells transfected with pCTR, LTco or LTco$^{\Delta}$ using Western blotting.

(B) Western blotting of LT in His-tagged IP of cells expressing LTco or LTco$^{\Delta}$. pCTR was used as a negative control.

(C) Detection of trLT in His-tagged IP from cells expressing pCTR, LT339 or LT339$^{\Delta}$.

(D) Analysis of RB-LT interaction in LT-IP from cells transfected with LT339 or LT339^{E216K}.

WCL, whole cell lysate; IP, immunoprecipitation.

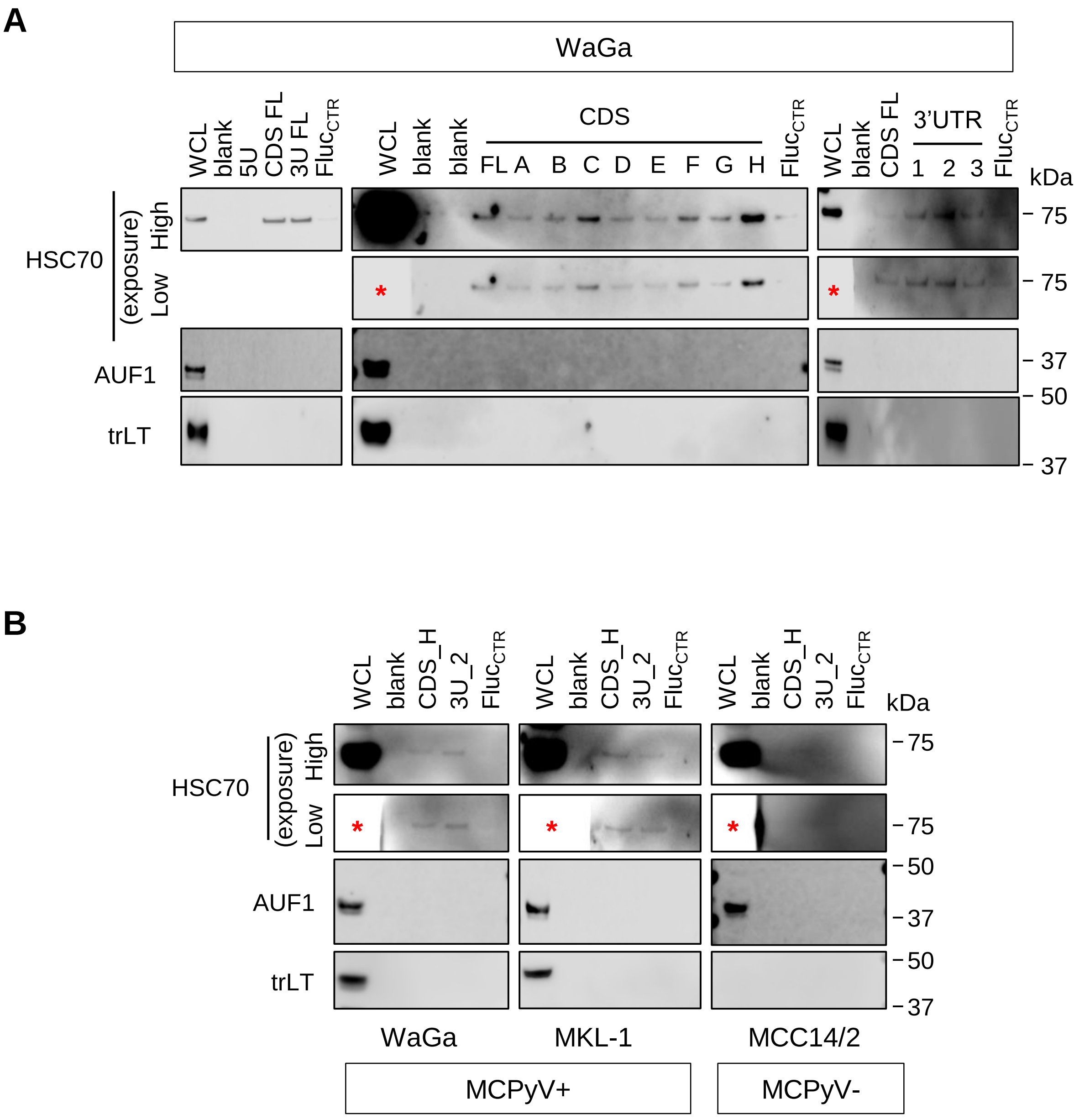

Figure S3. Evaluation of *DICER1* mRNA interaction with HSC70, AUF1 and LT using biotin pull-down assays, related to Figure 3.

(A) The same experiments and blots described in Figure 3E are shown here in high and low exposure. The panel also shows detection of AUF1.

(B) Biotin pull-down assays were repeated using CDS_H and 3U_2 probes in both MCPyV+ and MCPyV- MCC cell lines. $Fluc_{CTR}$ was used as a negative control. WCL, whole cell lysate; blank, empty lane. * Highly abundant protein was blocked during exposure. trLT, truncated LT.

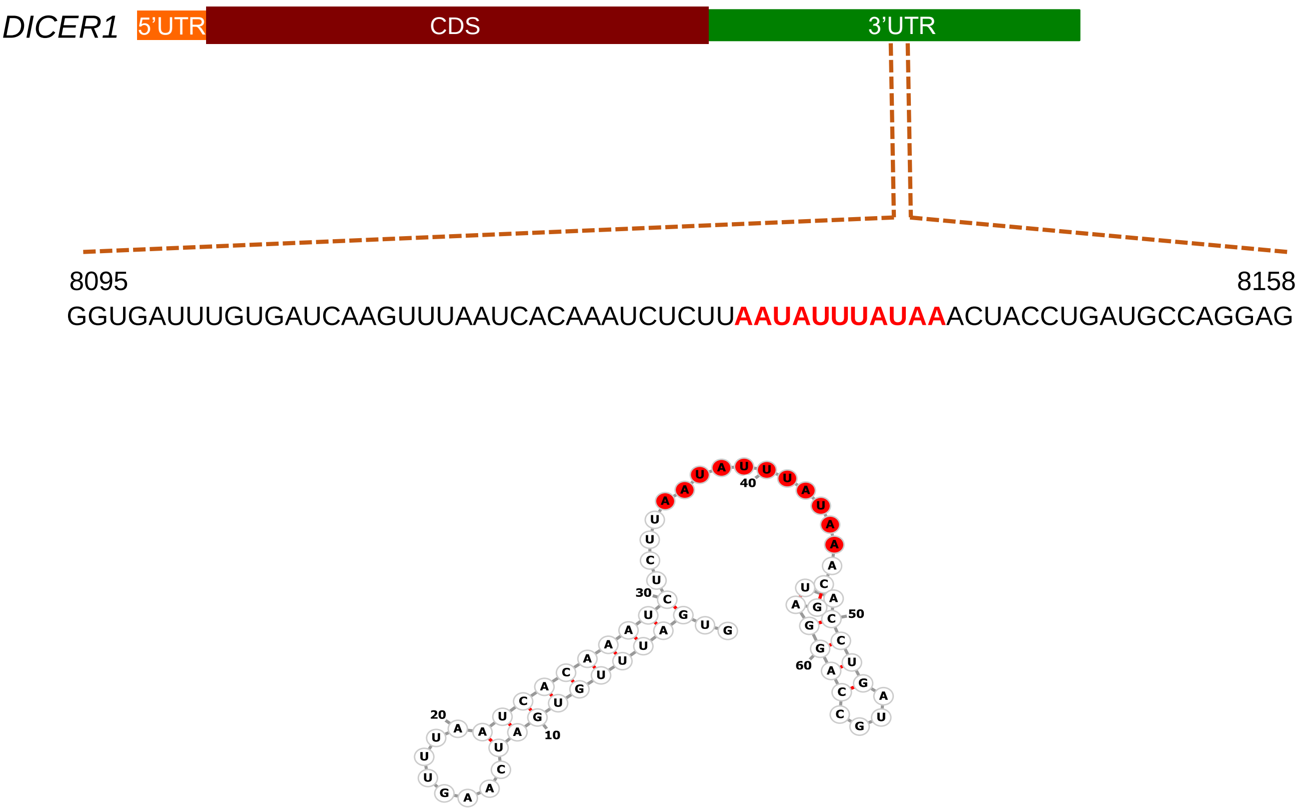

Figure S4. Analysis of the secondary structure of the ARE region in the 3'UTR of *DICER1*, related to Figure 4.

Upper: Schematic illustration of *DICER1* mRNA and the sequence (nt 8095-8158) in the 3'UTR that was used for RNA secondary structure analysis. The ARE sequence is highlighted in red.

Lower: Secondary structure of the sequence predicted by RNAfold. The ARE motif is indicated in red.

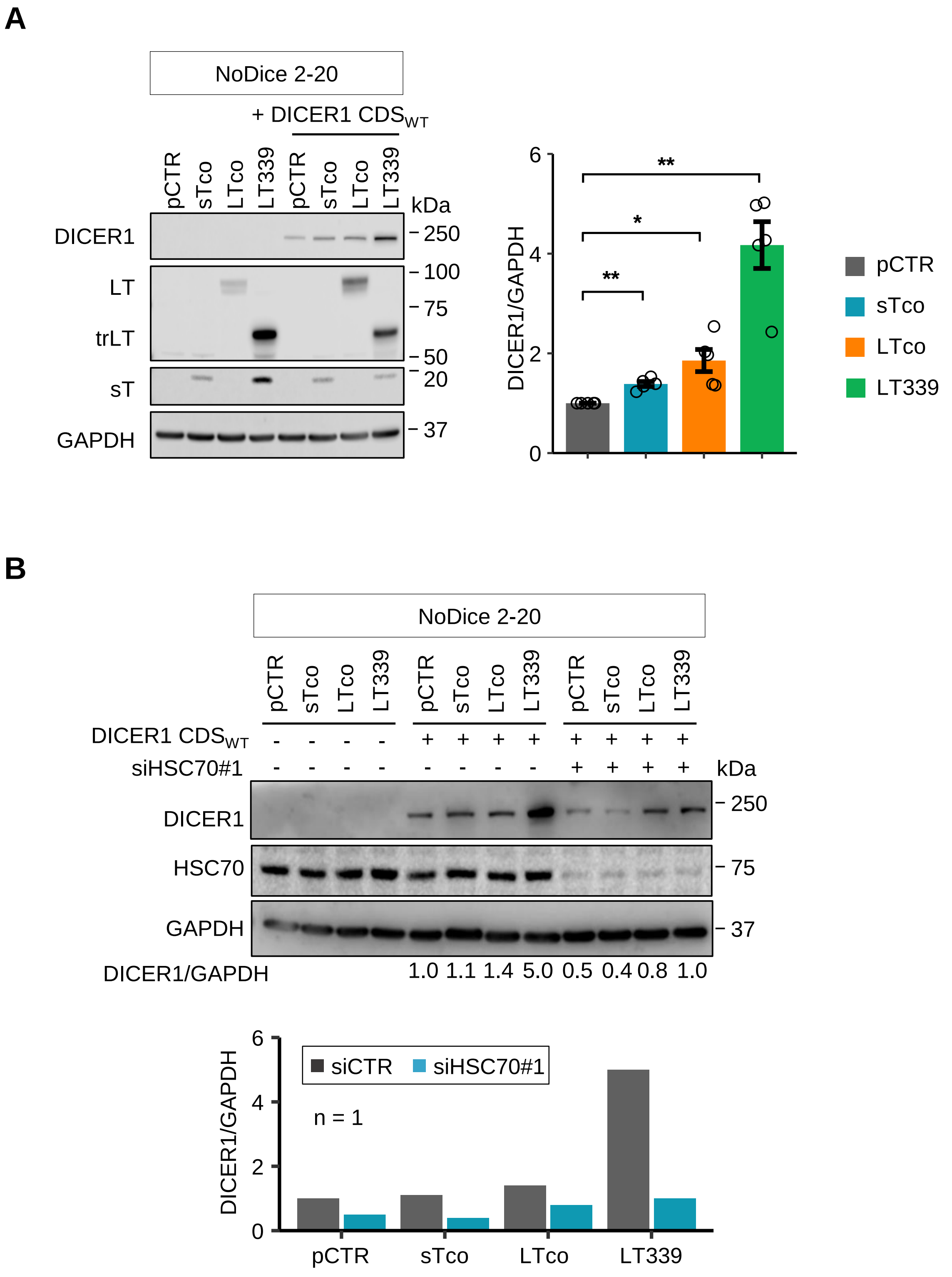

Figure S5. The effect of MCPyV T-antigens and HSC70 on exogenous DICER1 expression without 3'UTR, related to Figure 5. (A) Western blot analysis of NoDice 2-20 cells transfected with various T-antigens with and without DICER1 CDS_{WT}. *Left*: Representative Western blots showing the transfection efficiency and the effect of T-antigens on exogenous DICER1 expression. *Right*: Quantification of exogenous DICER1 expression normalized to GAPDH (n = 5). **p*<0.05, ***p*<0.01 by paired t-test and corrected with "Benjamini–Hochberg" method.

(B) The same experiments were repeated with and without silencing of HSC70. *Upper*: Western blots showing the effect of HSC70 silencing on exogenous DICER1 expression. The numbers and bar plot below the Western blots refer to DICER1/GAPDH ratios normalized to cells transfected with pCTR, *DICER1* CDS_{WT} and siCTR.
